# Identification of Genes Whose Expression Profile Is Associated with Non-Progression towards AIDS Using eQTLs

**DOI:** 10.1371/journal.pone.0136989

**Published:** 2015-09-14

**Authors:** Jean-Louis Spadoni, Pierre Rucart, Sigrid Le Clerc, Daniëlle van Manen, Cédric Coulonges, Damien Ulveling, Vincent Laville, Taoufik Labib, Lieng Taing, Olivier Delaneau, Matthieu Montes, Hanneke Schuitemaker, Josselin Noirel, Jean-François Zagury

**Affiliations:** 1 Chaire de Bioinformatique; Laboratoire Génomique, Bioinformatique, et Applications (EA 4627), Conservatoire National des Arts et Métiers, Paris, France; 2 Department of Experimental Immunology, Sanquin Research, Landsteiner Laboratory, and Center for Infectious Diseases and Immunity Amsterdam (CINIMA), Academic Medical Center, University of Amsterdam, Meibergdreef 15, 1105 AZ, Amsterdam, The Netherlands; 3 Crucell Holland B.V., Archimedesweg 4–6, 2333 CN, Leiden, The Netherlands; 4 Département de Génétique et Développement, Faculté de Médecine, Université de Genève, Switzerland; South Texas Veterans Health Care System and University of Texas Health Science Center at San Antonio, UNITED STATES

## Abstract

**Background:**

Many genome-wide association studies have been performed on progression towards the acquired immune deficiency syndrome (AIDS) and they mainly identified associations within the *HLA* loci. In this study, we demonstrate that the integration of biological information, namely gene expression data, can enhance the sensitivity of genetic studies to unravel new genetic associations relevant to AIDS.

**Methods:**

We collated the biological information compiled from three databases of expression quantitative trait loci (eQTLs) involved in cells of the immune system. We derived a list of single nucleotide polymorphisms (SNPs) that are functional in that they correlate with differential expression of genes in at least two of the databases. We tested the association of those SNPs with AIDS progression in two cohorts, GRIV and ACS. Tests on permuted phenotypes of the GRIV and ACS cohorts or on randomised sets of equivalent SNPs allowed us to assess the statistical robustness of this method and to estimate the true positive rate.

**Results:**

Eight genes were identified with high confidence (*p* = 0.001, rate of true positives 75%). Some of those genes had previously been linked with HIV infection. Notably, *ENTPD4* belongs to the same family as *CD39*, whose expression has already been associated with AIDS progression; while *DNAJB12* is part of the HSP90 pathway, which is involved in the control of HIV latency. Our study also drew our attention to lesser-known functions such as mitochondrial ribosomal proteins and a zinc finger protein, ZFP57, which could be central to the effectiveness of HIV infection. Interestingly, for six out of those eight genes, down-regulation is associated with non-progression, which makes them appealing targets to develop drugs against HIV.

## Introduction

More than fifteen genome-wide association studies (GWAS) have been conducted on AIDS since the seminal GWAS on HIV-1 progression in 2007 [1,2]. They mainly revealed associations in the region of the chromosome 6 *HLA* loci [1,3,4], in particular a single nucleotide polymorphism (SNP) in the *HCP5* gene, rs2395029. This SNP is in complete linkage disequilibrium with the *HLA-B5701* allele, already identified by several candidate-gene studies for its role in non-progression and the control of viral load [5–7]. Candidate gene studies also contributed to the discovery of another important polymorphisms, *CCR5-Δ32* [8–10].

Most of the genetic association studies on AIDS have relied on endpoints such as viral load at setpoint or time to reach a clinical symptom (e.g. CDC AIDS 1993 or death). The Genetics of Resistance to Immunodeficiency Virus (GRIV) cohort, composed of extreme phenotypes, non-progressors [11–13] and rapid progressors [14], is different since it relies on a case-control analysis.

For many human traits and diseases including AIDS, a substantial portion of heritability remains unexplained [15,16]. Strategies to increase the number of novel findings are being developed, including rare variants, facilitated by sequencing, or meta-analyses but with limited success to date [17].

Another approach that has received considerable attention is the use of pathway-based association tests that aim to look for an enrichment of associations in sets of genes within the same biological pathway [18–20]. Genetic association studies on AIDS have been described for specific pathways [21], but a systematic pathway analysis has yet to be performed. Increasingly popular, expression quantitative trait loci (eQTLs) quantify the relationship between genetic polymorphisms and gene transcription [22,23]. It has been proposed that eQTL are of the utmost important in the development of pathological traits [24,25].

In the present work, we have developed a novel, general-purpose pipeline based on the use of several eQTL databases as filters to preselect so-called functional SNPs. We used the databases “GHS_Express” by [26], “Gene Expression Analysis Based on Imputed Genotype” by [27], and “Genevar” by the Sanger Institute [28–30]. These databases correlate each SNP with gene expression in a specific cell line. We preselected the SNPs exhibiting the most significant *p*-values in each database, and to further warrant a prominent functional impact on gene expression, we selected the ones concurrently found in two or three databases. This restricted set of candidate functional SNPs was tested for genetic associations with AIDS using the GRIV cohort of extreme progression to AIDS [4,11–14] and using the Amsterdam Cohort Studies (ACS) cohort for replication [31]. As an added benefit, the eQTL databases allow us to directly associate gene expression levels with AIDS progression.

This unique approach selects a few hundred candidate SNPs to be tested for genetic association with AIDS, on the sole basis of their functional impact on gene expression, and it also differs from past candidate gene studies [32] since there was no gene pre-selection based on specific knowledge of AIDS biology. In this article, we describe how this approach was successful in unravelling novel, statistically-significant associations with biological activities particularly relevant to HIV-1 infection.

## Results

**Fig 1:**
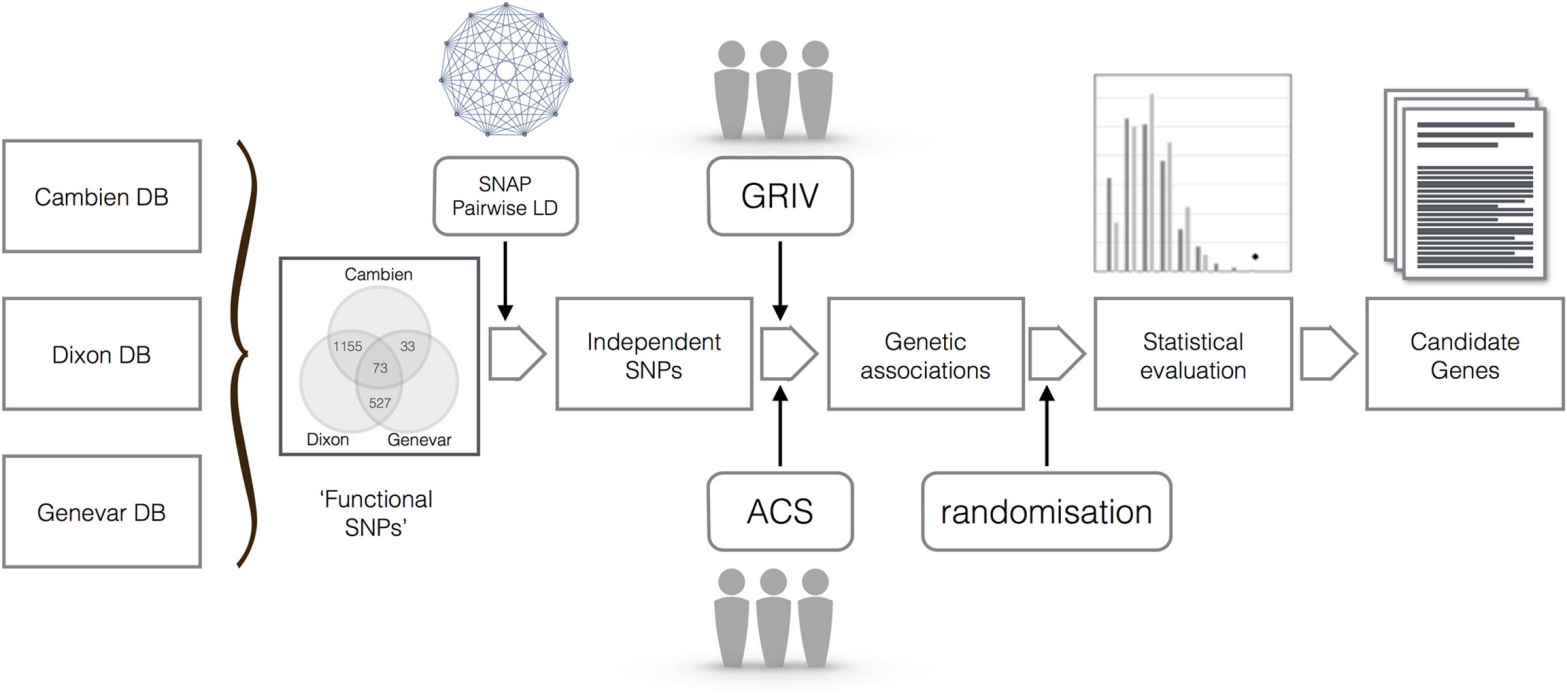
Schematic summary of our methodology. The data from three databases are integrated to provide us with functional SNPs likely to be associated with changes in gene transcription in the tissue of interest. Using the SNAP Pairwise LD server, we only kept independent SNPs by removing superfluous SNPs that were in linkage disequilibrium (*r* ^2^ ≥ 0.2). Among those SNPs, associations with slow and non-progression towards AIDS are sought and replicated. Randomisations are carried out in order to evaluate the statistical robustness of our results. Finally, the genetic associations are used to link progression to AIDS and gene expression in candidate genes.

Fig 1 shows a schematic overview of how we integrated eQTL data and progression towards AIDS in two cohorts to draw new genetic associations.

### Functional SNPs

We determined the modes of the associations between SNPs and gene transcription levels as described in the Methods section and found: 16,000 additive associations, 2173 recessive associations, 2050 dominant associations, 1133 overdominant associations, 3491 additive-or-dominant associations and 682 additive-or-recessive associations. Ambiguous modes were considered dominant for the remainder of the study, given the overwhelming majority of associations is additive.

Using the threshold *p* = 10^−4^ and the additive mode for eQTLs, we obtained a set of 1788 SNP/gene pairs corresponding to 1706 distinct SNPs and 567 distinct genes. The set of SNP/gene pairs is made of 73 pairs common to all three databases, 33 pairs common to Cambien and Genevar only, 527 pairs common to Dixon and Genevar only and 1155 pairs common to Cambien and Dixon only.

Linkage disequilibrium between those SNPs was removed. Pairs of SNPs with more than *r* ^2^ ≥ 0.2 were identified using the Broad Institute’s ‘SNAP Pairwise LD’. (See S2 File for details).

Such pairs define a ‘linkage disequilibirum graph’, where SNPs are vertices and edges connect SNPs in linkage disequilibrium. The connected components of this graph are calculated and a representative SNP is chosen for each connected component (for convenience’s sake, we chose the SNP with the lowest ID in dbSNP). Generally, though not always, consistent groups of genes are regulated among the SNPs within a connected component. We obtain a set of 655 independent SNPs (see S1 Table).

### Genetic associations with AIDS progression

Of the 655 SNPs selected above, *N* = 654 could be imputed in GRIV and ACS cohorts and their association with slow progression or non-progression to AIDS could be sought in GRIV and replicated in ACS as described in the Methods section. Given that there was a small number of SNPs to be tested and that we resorted to replication, a *p*-value of *α* = 0.05 was used both for the association in the GRIV cohort and for the replication in the ACS cohort. With this choice, a low type-I error was expected.

### Genetic associations

Nine out of the 654 SNPs are associated with slow and non-progression towards AIDS (see Table 1 and S1 File for the Q-Q plots). Which is more than the number of associations expected by chance. We recalculated all the *r* ^2^ values for the associations on chromosome 6 in the DESIR cohort. All associations are at linkage equilibrium but one pair: rs3130350/rs3749971 (*r* ^2^ = 0.6, distance 985 kbp). These SNPs were not reported by the SNAP Pairwise LD server because they are more than 500 kbp apart. The final number of independent associations is 8.

**Table 1:** List of the significant associations (*p* ≤ 0.05) with slow and non-progression. Alleles, allele frequencies (AF), positional data and genetic modes are provided with the results of the statistical inferences. Opposite signs for the β coefficients are required for an association to be replicated in the GRIV (non-progression) and ACS cohorts (time to AIDS93).

SNP	Alleles Ref/Alt		AF (Alt)	Chromosome/position		Mode	*β* (GRIV)	*p* (GRIV)	*β* (ACS)	*p* (ACS)	Allele associated with non-progression
**732563**	T	C	53%	8	23,488,013	additive	-0.23	0.029	0.21	0.029	T
**732563**	T	C	53%	8	23,488,013	recessive	-0.41	0.024	0.40	0.007	T
**2205418**	T	C	12%	21	28,657,885	recessive	-2.24	0.038	2.81	1.5·10^–4^	T
**2241335**	C	T	45%	7	134,294,616	dominant	0.44	0.015	-0.34	0.034	T
**2242229**	G	T	78%	17	75,246,420	recessive	0.49	0.003	-0.33	0.025	T
**2921446**	C	A	66%	10	72,691,727	dominant	0.53	0.037	-0.48	0.024	A
**3130350**	G	T	7%	6	30,360,062	additive	-0.66	0.019	0.51	0.001	G
**3130350**	G	T	7%	6	30,360,062	dominant	-0.73	0.015	0.42	0.010	G
**3130501**	A	G	77%	6	31,168,676	dominant	-0.78	0.021	0.81	0.038	A
**3749971** *	G	A	6%	6	29,374,998	dominant	-0.67	0.036	0.37	0.031	G
**4714580**	A	G	83%	6	42,206,820	recessive	-0.32	0.049	0.33	0.045	A

(*) Note that rs3749971 is in linkage disequilibrium with rs3130350 and is therefore not considered an independent finding in our statistics (see text).

### Statistical significance: estimation of the sensitivity

**Fig 2:**
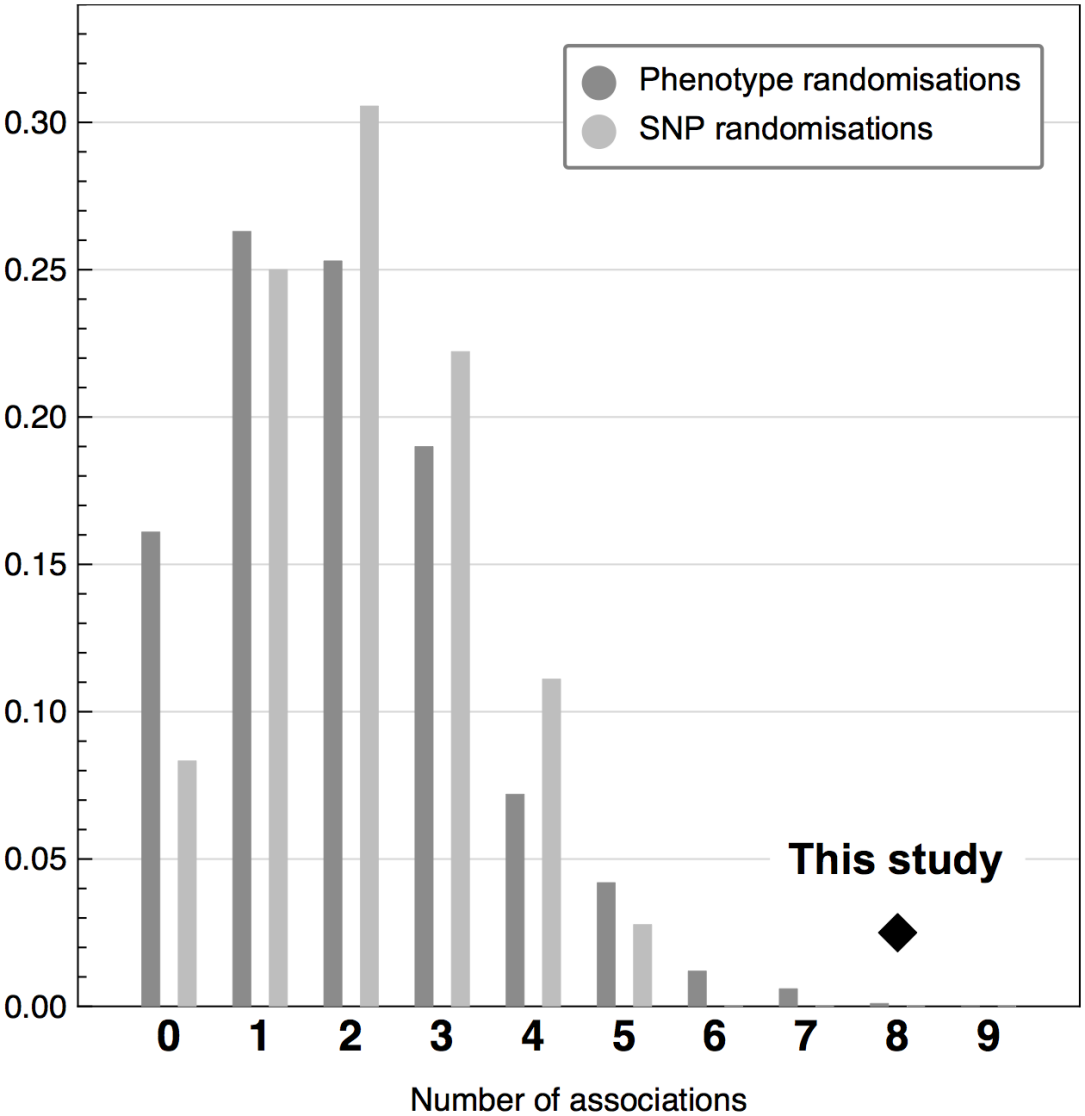
Statistical significance of our associations. Histogram of the number of SNPs that pass the significance criterion for this study using phenotype and SNP randomisations. These results provide us with a way to estimate the sensitivity of our study (diamond): it would be extremely unlikely for our eight independent findings to arise by chance alone (*p* = 0.001).

To demonstrate that the number of significant hits really arises from associations between polymorphisms and the phenotypes, we carried out two sets of randomisations: (1) phenotype randomisation to show that the choice of the functional SNPs is itself not sufficient to warrant significant hits, (2) SNP randomisation to show that neither is the spectrum of phenotypes is. Those randomisations come with the added benefit that we can estimate the false discovery rate and its distribution (hence, of the sensitivity of our method). We carried out 1000 phenotype randomisations and 36 SNP randomisations (12 of which are sampled from the eQTL databases). The results are shown in Fig 2.

The phenotype randomisations allow us to estimate two useful, complementary measures from the distribution: the fraction of expected false positives (‘sensitivity’) and the likelihood of obtaining as many independent SNPs as we did or more by chance alone (*p*-value). The sensitivity (true positives) is estimated at 75% (in other words 25% false discovery rate) with a 95% credible interval of 38% to 100%. Under the null hypothesis, the probability of obtaining as many independent SNPs as we did or more is *p* = 0.001.

Although their number is far lower than that of phenotype randomisations (due to technical reasons, see Materials and Methods), SNP randomisations give results that are well aligned with phenotype randomisations: the location and spread of the distributions cannot be distinguished.

### Regulated genes and biological significance

We mapped the 8 SNPs back to the corresponding genes of the SNP/gene pairs identified earlier and the direction of the correlation with each gene’s expression. This allowed us to associate gene expression with slow or non-progression to AIDS. The expression of the following genes therefore could be important in progression towards AIDS: *CCT8*, *DNAJB12*, *ENTPD4*, *GUCA1B*, *HCG27*, *MRPS7*, *MRPS10*, *SLC35B4* and *ZFP57*. No additional gene was identified by considering the connected components of the LD graph. The results are presented in Table 2.

**Table 2:** List of SNP/gene pairs associated with AIDS progression. The genetic association is linked back to its association with gene expression levels to provide an association between transcription levels (we use the word ‘regulation’ for convenience’s sake) and AIDS progression.

SNP	Chromosome	Gene	Gene function	Association with non-progression
**732563**	8	*ENTPD4*	ectonucleoside triphosphate diphosphohydrolase 4	Down-regulation
**2205418**	21	*CCT8*	chaperonin containing TCP1, subunit 8 (theta)	Down-regulation
**2241335**	7	*SLC35B4*	solute carrier family 35 (UDP-xylose/UDP-N-acetylglucosamine transporter), member B4	Up-regulation
**2242229**	17	*MRPS7*	mitochondrial ribosomal protein S7	Up-regulation
**2921446**	10	*DNAJB12*	DnaJ (Hsp40) homolog, subfamily B, member 12	Down-regulation
**3749971/3130350**	6	*ZFP57*	ZFP57 zinc finger protein	Down-regulation
**3130501**	6	*HCG27*	HLA complex group 27 (non-protein coding)	Down-regulation
**4714580**	6	*GUCA1B*	guanylate cyclase activator 1B (retina)	Down-regulation
**4714580**	*6*	*MRPS10*	mitochondrial ribosomal protein S10	Down-regulation

(*) Note that rs3749971 is in linkage disequilibrium with rs3130350 and is therefore not considered an independent finding (see text).

The *ENTPD4* gene codes for a protein involved in the metabolism of purines and pyrimidines. Our results suggest that down-regulation of *ENTPD4* is associated with a slower progression to AIDS. It is known that CD39, a member of the ENTPD family, is the dominant immune system ectonucleotidase that hydrolyses extracellular ATP and ADP into AMP at the sites of immune activation. A previous study indicated that a down-regulated *CD39* expression in CD4 T cells was associated with a slower progression to AIDS [21,33]. Interestingly, the *ENTPD4* gene is among the genes significantly up-regulated (6-fold up-regulation) during antigen presentation in CD4 T cells by the presence of HIV’s gp120/V3 peptides [34]. *ENTPD4* is also expressed in B lymphocytes [35] and GTEx data support a weak association between rs2241335 and rs2241336 polymorphism and *ENTPD4* expression levels (*p* = 0.1, *N* = 42) in the cell line of EBV-transformed lymphocytes [22].

Interestingly, two nuclear genes coding for mitochondrial ribosomal proteins were found in our study (*MRPS7* on chromosome 17 and *MRPS10* on chromosome 6). Somewhat surprisingly, a *lower* transcription of *MRPS10* but a *higher* transcription of *MRPS7* are associated with slow and non-progression towards AIDS. Although the involvement in AIDS progression of those genes has never been reported, the implication of mitochondria in AIDS pathogenesis has been suggested by several studies in the past. For example, genetic associations between mitochondrial haplotypes and AIDS progression have been reported [36,37]. *MRPS12* was also among 185 genes predictive of HIV-1 resistance and 29 infection information exchanger genes [38]; as a consequence, Huang and colleagues speculated that *MRPS12* could be important for the coordination of HIV infection. Furthermore, gene set analyses have suggested that mitochondria could be key in the immune response against HIV infections even though the exact pathways (energy metabolism, cell apoptosis or cell cycle dysregulation) are yet to be identified [39]. MRPS10 interacts with a number of proteins involved in HIV aetiology (see S1 File). An association between *MRPS7*’s expression and rs2242229 is also reported in the Geuvadis database (*p* = 3.4×10^−23^) [23].

The identification of *DNAJB12* as a potentially important gene is reminiscent of the observation that the HSP40/DNAJ family proteins play a role in infection of various viruses. Urano and colleagues identified DNAJ/HSP40B6 as a potential regulator of HIV-1 replication [40]. It is an interesting finding considering the attention that another chaperone, namely HSP90, has recently attracted: HSP90 could promote infectiousness of HIV by controlling HIV reactivation from latency [41] and several inhibitors of HSP90 are currently in clinical development [42]. This is consistent with our observation that a lower expression of *DNAJB12*, which is part of the HSP90 pathway [43], correlates with slow or non-progression towards AIDS. DNAJB12 also interacts with many proteins known to be associated with HIV infection: among the twelve protein-protein interactions reported by InnateDB [44], eight proteins (DSTN, EGFR, HSPA8, MME, MYC, SGTA and UBC) are also found in the HIV-1 Human Interaction Database [45] (see S1 File). The association of low expression of chaperone-coding gene *CCT8* with slow- and non-progression also points to a role of chaperones in promoting HIV infection; also of interest is the observation that CCT8 is one of the proteins to be differentially regulated in synaptosomal isolates from HIV/gp120 transgenic mice [46]; an association between *CCT8*’s expression and rs2205418 is also reported in the Geuvadis database (*p* = 1.7×10^−8^) [23].

*ZFP57* is a transcriptional regulator involved in DNA methylation and genomic imprinting during development but its gene expression also occurs at in adult peripheral blood cells [47]. A lower transcription correlates with slow or non-progression towards AIDS. As reported by Plant et al., SNPs associated with differential transcription of this gene have already been highlighted as associated with AIDS progression [4,47,48]. The impact of ZFP57 could be due to the promotion of viral latency through hypermethylation [47] or through its interaction with TRIM28, which was shown to enhance HIV infections in model cell lines [49].

The gene *HCG27* (HLA complex group 27) is a non-protein coding gene; though *HLA* genes have been consistently been associated with AIDS progression [2,50], the role of this gene (as well as that of other candidate genes revealed in this study, such as *SLC35B4*, *GUCA1B*) is difficult to evaluate to the best of our knowledge. Note that *GUCA1B* could be a passenger finding: indeed, it is the second of two genes whose expression correlates with the rs4714580 polymorphism, the first being *MRPS10*, which might be the causal gene.

### Associations with rapid progression towards AIDS

Genetic associations with rapid progression towards AIDS were sought among the list of 654 functional SNPs. Our randomisation tests demonstrated that the associations were not statistically significant (see S1 File). This demonstrates that these randomisation procedures act as effective safeguards against spurious associations.

## Discussion

Genomewide association studies have successfully allowed the confident discovery of many factors involved in human diseases. However, they haven’t yet told the whole story. In AIDS, they have mostly yielded associations in the *HLA* region and in relation with the *CCR5* region [12]. Still, there obviously is a gap between our current ability to detect genetic associations and our capacity to predict the risk based on genetics alone; the ‘missing heritability’ hints that there remain difficult-to-identify genetic markers [15,51]. Larger cohorts are used to overcome this [17] but another route consists in integrating data from various sources. This study provides a successful implementation of the second solution.

The rationale behind our work is that the integration of eQTL data provides us with a reliable SNP/gene map, not without similarities with the concept of ‘eSNP’ (genetic variants directly associated with higher or lower transcript expression levels), which is more likely to point towards functional and causal factors. Using three gene expression databases (GHS_Express, Gene Expression Analysis Based on Imputed Genotype and Genevar), we could identify polymorphisms more likely to play a functional role. Our approach is substantiated by the number of SNP/gene pairs confirmed in the GTEx and Geuvadis DBs. The randomisations involving SNPs sampled from the eQTL databases highlight the value of integrating data from several databases.

We looked for genetic associations in GRIV and replicated those in ACS and found eight independent SNPs significantly associated with slow or non-progression to AIDS. An important facet of this study was the evaluation of the statistical robustness of our findings, which confidently supports a significant of positive associations.

eQTL databases were not only essential in the preparation of a carefully-selected set of functional SNPs, they also were instrumental in identifying candidate genes whose expression profiles could be more directly associated with the AIDS progression phenotypes: *ENTPD4*, *CCT8*, *SLC35B4*, *MRPS7*, *MRPS10*, *DNAJB12*, *ZFP57*, *HCG27* and *GUCA1B*. Overall, we have found in the existing literature and in published datasets compelling biological grounds for the possible implication of the genes identified in this study in progression to AIDS. Interestingly, for six out of those eight genes, down-regulation is associated with non-progression, which makes them appealing targets to combat HIV infections. Importantly, our study revealed a number of yet uninvestigated candidate genes, which can further our understanding of AIDS infection and AIDS progression as well as facilitate the discovery of new drugs.

Researchers working on other diseases could easily apply this method to their own genome-wide datasets. The set of 655 functional SNPs is provided as S1 Table and is available from http://www.griv.org/functSNPs/.

## Materials and Methods

### Sets of functional SNPs

In this work, we investigated associations between gene expression and AIDS progression. The associations were sought through the selection of ‘functional SNPs’, known to be associated with changes in gene transcription levels; informally, we can define a functional SNP as a SNP likely to have a direct biological action through gene expression. We achieved this by integrating the data obtained from three separate mRNA-expression databases: GHS_Expression (hereafter referred to as the ‘Cambien’ database) [26], Gene Expression Analysis Based on Imputed Genotype (hereafter referred to as the ‘Dixon database) [27] and Genevar [28–30]. (See S2 File for details).

#### Data integration

Formally, a SNP *s* is considered functional if it meets the following criterion: there exists a gene *g* such that the genotype of *s* consistently and significantly (reported *p*-value is less than 10^−4^) correlates with the transcription levels of the gene *g* in at least two of the three databases Cambien, Dixon and Genevar. This selection warrants high confidence in the selection. The mode (additive) must be coherent throughout the datasets and the correlation must be consistent including across different probes within a single dataset. The set of functional SNPs is a list of elements ({*S*, *s*}, *g*, *m*, *d*, *D*), where {*S*, *s*} is a biallelic SNP (defined as a major/minor pair of alleles), *g* is a gene, *m* is the mode in which the allele *s* is associated with an altered expression of the gene *g*, *d* is the direction of regulation (+ or-) and *D* the datasets where the regulation is observed (Cambien, Dixon or Genevar).

The threshold *p* < 10^−4^ was partly constrained by the fact that the Cambien database only reports associations for which the *p*-value is less than 10^−4^ already. Had the data been complete, other choices of threshold could have been made, provided the significance of the results could be ascertained using the randomisations described below.

### Cohorts

#### The Genomics of Resistance to Immunodeficiency Virus Cohort (GRIV) and the French control group

The GRIV cohort, established in 1995 in France, is a collection of DNA samples used to identify host genes associated with slow progression and with rapid progression to AIDS [7,10,52]. The study was reviewed and approved by the institutional review board of Hôpital Saint-Louis (Paris, France) before the study began. All participants provided written informed consent. Only white individuals of European descent living in France were eligible for enrolment to reduce confounding effects by population substructure. These criteria limit the influence of the ethnic and environmental factors (all subjects live in a similar environment and are infected by HIV-1 subtype B strains) and put an emphasis on the genetic make-up of each individual in determination of long term non-progression (NP) to AIDS. The NP group (*n* = 270) was composed of 200 males and 70 females aged at inclusion from 19 to 62 (mean age 35). We used the Data from an Epidemiological Study on Insulin Resistance Syndrome (DESIR) program as a control group. (See S2 File for details).

#### The Amsterdam Cohort Studies Cohort (ACS)

The ACS cohort was composed of 316 HIV-1 homosexual men. The study was reviewed and approved by the AMC Medical Ethics Committee. All participants provided written informed consent. This cohort was established to follow the course of HIV-1 infection using various endpoints related to HIV-1 infection and AIDS [31,53].

### Processing genomic data

We excluded individuals who were related and outliers based on population stratification. SNPs were excluded when, within the control group, they were out of Hardy-Weinberg equilibrium, when the minor allelic frequency was less than 1% or when missing data were greater than 2%. Individuals with more than 5% missing data or with high heterozygosity were excluded.

In order to identify all the known SNPs in LD with our selected list of SNPs, present in the HapMap database, we imputed all SNPs in the GRIV, ACS and control subjects using the 1000 Genomes phase I data [54]. Only the SNPs reliably imputed were retained.

The SNPs associated with non-progression towards AIDS were sought in the GRIV cohort and then replicated in ACS. In order to be replicated an association must be consistent in terms of its genetic mode and effect direction.

For each functional SNP, we computed the *p*-values of the association in the GRIV cohort either with non-progression or with rapid progression using a standard case-control analysis (non-progression vs control and rapid progression vs control). All modes (dominant, recessive, and additive) were tested. Sex and the first two stratification axes were included as covariates. The significance threshold for an association with progression was set at *α* = 0.05.

For each functional SNP, we computed the *p*-values of the association in the ACS cohort with progression using the (censored) variable ‘time to AIDS 1993 after HIV-1 infection’ [10]. The first two stratification axes were included as covariates (sex was not included as all subjects are male). (See S2 File for details).

### Randomisation tests

Though the method described in this paper used expression data to investigate associations between gene regulation and phenotypes, we assessed the significance of our findings at the SNPs’ level. Indeed, there currently does not exist a method to randomise expression data alongside genotypes. Therefore, the significance really tests the usability and robustness of the concept of ‘functional SNP’ we have used in this study. The significance of our findings was testing using two randomisation procedures: phenotype randomisation and SNP randomisation.

#### Phenotype randomisation

In GRIV, the NP and control phenotypes were randomised using GNU R’s *sample* function. In ACS, the ‘time to AIDS 1993’ variable was similarly randomised using GNU R’s *sample* function; the corresponding censored status was carried along during randomisation.

#### SNP randomisation

In order to avoid any bias, 36 sets of SNPs comparable to the set of functional SNPs described above were selected for the SNP randomisation. SNP sets had to be comparable in terms of allele frequency, genotyping/imputation ratio, linkage disequilibrium, and distance to nearest gene. Other factors (sex, stratification principal components, outcome) were maintained unaltered. Only 36 sets were produced: (1) given the constraints, the number of sets is limited by nature, (2) SNP randomisation is computationally expensive than phenotype randomisation, (3) the distribution resulting from SNP randomisation is used to corroborate the distribution obtained using more extensive phenotype randomisation. (See S2 File for details).

## Supporting Information

S1 File Supporting Results. Which eQTL database can the functional SNPs traced back to? Q-Q plots for the analyses. Negative results for rapid progression. Interactions between the identified genes and AIDS/HIV.
(PDF)

S2 File Supporting Methods. Mode in the Cambien dataset. Additional details about the cohorts. Workflow for preprocessing genomic data and testing the association with slow or non-progression. Parameters for linkage disequilibrium. Details about the SNP randomisation.
(PDF)

S1 Table List of functional SNPs. List of SNPs which with consistent behaviour in the three eQTL databases used in this work.
(XLSX)

## References

[1] Fellay J, Shianna KV, Ge D, Colombo S, Ledergerber B, Weale M, et al A whole-genome association study of major determinants for host control of HIV-1. Science. 2007;317: 944–947. 10.1126/science.1143767 17641165 PMC1991296

[2] Limou S, Zagury J-F. Immunogenetics: Genome-wide association of non-progressive HIV and viral load control: *HLA* genes and beyond. Front Immunol. 2013;4: 118 10.3389/fimmu.2013.00118 23750159 PMC3664380

[3] Dalmasso C, Carpentier W, Meyer L, Rouzioux C, Goujard C, Chaix M-L, et al Distinct genetic loci control plasma HIV-RNA and cellular HIV-DNA levels in HIV-1 infection: the ANRS Genome Wide Association 01 study. PLoS ONE. 2008;3: e3907 10.1371/journal.pone.0003907 19107206 PMC2603319

[4] Limou S, Le Clerc S, Coulonges C, Carpentier W, Dina C, Delaneau O, et al Genomewide association study of an AIDS-nonprogression cohort emphasizes the role played by *HLA* genes (ANRS Genomewide Association Study 02). J Infect Dis. 2009;199: 419–426. 10.1086/596067 19115949

[5] Carrington M, Nelson GW, Martin MP, Kissner T. *HLA* and HIV-1: heterozygote advantage and *B*35-Cw*04* disadvantage. Science. 1999 10.1126/science.283.5408.1748 10073943

[6] Hendel H, Caillat-Zucman S, Lebuanec H, Carrington M, O'Brien S, Andrieu JM, et al New class I and II *HLA* alleles strongly associated with opposite patterns of progression to AIDS. J Immunol. 1999;162: 6942–6946. 10352317

[7] Flores-Villanueva PO, Hendel H, Caillat-Zucman S, Rappaport J, Burgos-Tiburcio A, Bertin-Maghit S, et al Associations of *MHC* ancestral haplotypes with resistance/susceptibility to AIDS disease development. J Immunol. 2003;170: 1925–1929. 10.4049/jimmunol.170.4.1925 12574360

[8] Dean M, Carrington M, Winkler C, Huttley GA, Smith MW, Allikmets R, et al Genetic Restriction of HIV-1 Infection and Progression to AIDS by a Deletion Allele of the *CKR5* Structural Gene. Science. 1996;273: 1856–1862. 10.1126/science.273.5283.1856 8791590

[9] Samson M, Libert F, Doranz BJ, Rucker J, Liesnard C, Farber CM, et al Resistance to HIV-1 infection in caucasian individuals bearing mutant alleles of the CCR-5 chemokine receptor gene. Nature. 1996;382: 722–725. 10.1038/382722a0 8751444

[10] Winkler CA, Hendel H, Carrington M, Smith MW, Nelson GW, O'Brien SJ, et al Dominant effects of *CCR2-CCR5* haplotypes in HIV-1 disease progression. J Acquir Immune Defic Syndr. 2004;37: 1534–1538. 15602133 10.1097/01.qai.0000127353.01578.63

[11] Le Clerc S, Coulonges C, Delaneau O, Van Manen D, Herbeck JT, Limou S, et al Screening low-frequency SNPs from genome-wide association study reveals a new risk allele for progression to AIDS. J Acquir Immune Defic Syndr. 2011;56: 279–284. 21107268 10.1097/QAI.0b013e318204982b PMC3386792

[12] Limou S, Coulonges C, Herbeck JT, Van Manen D, An P, Le Clerc S, et al Multiple-cohort genetic association study reveals CXCR6 as a new chemokine receptor involved in long-term nonprogression to AIDS. J Infect Dis. 2010;202: 908–915. 10.1086/655782 20704485 PMC3601691

[13] Limou S, Delaneau O, Van Manen D, An P, Sezgin E, Le Clerc S, et al Multicohort genomewide association study reveals a new signal of protection against HIV-1 acquisition. J Infect Dis. 2012;205: 1155–1162. 10.1093/infdis/jis028 22362864 PMC3295605

[14] Le Clerc S, Limou S, Coulonges C, Carpentier W, Dina C, Taing L, et al Genomewide association study of a rapid progression cohort identifies new susceptibility alleles for AIDS (ANRS Genomewide Association Study 03). J Infect Dis. 2009;200: 1194–1201. 10.1086/605892 19754311

[15] Maher B. Personal genomes: The case of the missing heritability. Nature. 2008;456: 18–21. 10.1038/456018a 18987709

[16] O'Brien SJ, Nelson GW. Human genes that limit AIDS. Nat Genet. 2004;36: 565–574. 10.1038/ng1369 15167933

[17] McLaren PJ, Coulonges C, Ripke S, van den Berg L, Buchbinder S, Carrington M, et al Association study of common genetic variants and HIV-1 acquisition in 6,300 infected cases and 7,200 controls. PLoS Pathog. 2013;9: e1003515 10.1371/journal.ppat.1003515 23935489 PMC3723635

[18] Cantor RM, Lange K, Sinsheimer JS. Prioritizing GWAS results: A review of statistical methods and recommendations for their application. Am J Hum Genet. 2010;86: 6–22. 10.1016/j.ajhg.2009.11.017 20074509 PMC2801749

[19] Eleftherohorinou H, Wright V, Hoggart C, Hartikainen A-L, Jarvelin M-R, Balding D, et al Pathway analysis of GWAS provides new insights into genetic susceptibility to 3 inflammatory diseases. PLoS ONE. 2009;4: e8068 10.1371/journal.pone.0008068 19956648 PMC2778995

[20] Wang K, Li M, Hakonarson H. Analysing biological pathways in genome-wide association studies. Nat Rev Genet. 2010;11: 843–854. 10.1038/nrg2884 21085203

[21] Nikolova M, Carriere M, Jenabian M-A, Limou S, Younas M, Kök A, et al CD39/adenosine pathway is involved in AIDS progression. PLoS Pathog. 2011;7: e1002110 10.1371/journal.ppat.1002110 21750674 PMC3131268

[22] GTEx Consortium. The Genotype-Tissue Expression (GTEx) project. Nat Genet. 2013;45: 580–585. 10.1038/ng.2653 23715323 PMC4010069

[23] Lappalainen T, Sammeth M, Friedländer MR, 't Hoen PAC, Monlong J, Rivas MA, et al Transcriptome and genome sequencing uncovers functional variation in humans. Nature. 2013;501: 506–511. 10.1038/nature12531 24037378 PMC3918453

[24] Nicolae DL, Gamazon E, Zhang W, Duan S, Dolan ME, Cox NJ. Trait-associated SNPs are more likely to be eQTLs: annotation to enhance discovery from GWAS. PLoS Genet. 2010;6: e1000888 10.1371/journal.pgen.1000888 20369019 PMC2848547

[25] Kang M, Zhang C, Chun H-W, Ding C, Liu C, Gao J. eQTL epistasis: detecting epistatic effects and inferring hierarchical relationships of genes in biological pathways. Bioinformatics. 2015;31: 656–664. 10.1093/bioinformatics/btu727 25359893

[26] Zeller T, Wild P, Szymczak S, Rotival M, Schillert A, Castagne R, et al Genetics and beyond—the transcriptome of human monocytes and disease susceptibility. PLoS ONE. 2010;5: e10693 10.1371/journal.pone.0010693 20502693 PMC2872668

[27] Dixon AL, Liang L, Moffatt MF, Chen W, Heath S, Wong KCC, et al A genome-wide association study of global gene expression. Nat Genet. 2007;39: 1202–1207. 10.1038/ng2109 17873877

[28] Dimas AS, Deutsch S, Stranger BE, Montgomery SB, Borel C, Attar-Cohen H, et al Common regulatory variation impacts gene expression in a cell type-dependent manner. Science. 2009;325: 1246–1250. 10.1126/science.1174148 19644074 PMC2867218

[29] Stranger BE, Forrest MS, Clark AG, Minichiello MJ, Deutsch S, Lyle R, et al Genome-wide associations of gene expression variation in humans. PLoS Genet. 2005;1: e78 10.1371/journal.pgen.0010078 16362079 PMC1315281

[30] Stranger BE, Forrest MS, Dunning M, Ingle CE, Beazley C, Thorne N, et al Relative impact of nucleotide and copy number variation on gene expression phenotypes. Science. 2007;315: 848–853. 10.1126/science.1136678 17289997 PMC2665772

[31] Van Manen D, Delaneau O, Kootstra NA, Boeser-Nunnink BD, Limou S, Bol SM, et al Genome-wide association scan in HIV-1-infected individuals identifying variants influencing disease course. PLoS ONE. 2011;6: e22208 10.1371/journal.pone.0022208 21811574 PMC3141012

[32] Fellay J. Host genetics influences on HIV type-1 disease. Antivir Ther. 2009;14: 731–738. 10.3851/IMP1253 19812435 PMC2851194

[33] Chevalier MF, Weiss L. The split personality of regulatory T cells in HIV infection. Blood. 2013;121: 29–37. 10.1182/blood-2012-07-409755 23043072

[34] Morou AK, Porichis F, Krambovitis E, Sourvinos G, Spandidos DA, Zafiropoulos A. The HIV-1 gp120/V3 modifies the response of uninfected CD4 T cells to antigen presentation: mapping of the specific transcriptional signature. J Transl Med. 2011;9: 160 10.1186/1479-5876-9-160 21943198 PMC3203262

[35] Wilhelm M, Schlegl J, Hahne H, Gholami AM, Lieberenz M, Savitski MM, et al Mass-spectrometry-based draft of the human proteome. Nature. 2015;509: 582–587. 10.1038/nature13319 24870543

[36] Guzmán-Fulgencio M, Jiménez JL, García-Álvarez M, Bellón JM, Fernández-Rodriguez A, Campos Y, et al Mitochondrial haplogroups are associated with clinical pattern of AIDS progression in HIV-infected patients. J Acquir Immune Defic Syndr. 2013;63: 178–183. 23666137 10.1097/QAI.0b013e3182893f74

[37] Hendrickson SL, Hutcheson HB, Ruiz-Pesini E, Poole JC, Lautenberger J, Sezgin E, et al Mitochondrial DNA haplogroups influence AIDS progression. AIDS. 2008;22: 2429–2439. 19005266 10.1097/QAD.0b013e32831940bb PMC2699618

[38] Huang T, Xu Z, Chen L, Cai Y-D, Kong X. Computational analysis of HIV-1 resistance based on gene expression profiles and the virus-host interaction network. PLoS ONE. 2011;6: e17291 10.1371/journal.pone.0017291 21394196 PMC3048858

[39] Wu JQ, Dwyer DE, Dyer WB, Yang YH, Wang B, Saksena NK. Genome-wide analysis of primary CD4+ and CD8+ T cell transcriptomes shows evidence for a network of enriched pathways associated with HIV disease. Retrovirology. 2011;8: 18 10.1186/1742-4690-8-18 21410942 PMC3068086

[40] Urano E, Morikawa Y, Komano J. Novel Role of HSP40/DNAJ in the Regulation of HIV-1 Replication. J Acquir Immune Defic Syndr. 2013;64: 154–162. 24047968 10.1097/QAI.0b013e31829a2ef8

[41] Anderson I, Low JS, Weston S, Weinberger M, Zhyvoloup A, Labokha AA, et al Heat shock protein 90 controls HIV-1 reactivation from latency. Proc Natl Acad Sci USA. 2014;111: E1528–37. 10.1073/pnas.1320178111 24706778 PMC3992654

[42] Low JS, Fassati A. Hsp90: a chaperone for HIV-1. Parasitology. 2014;141: 1192–1202. 10.1017/S0031182014000298 25004926

[43] Cintron NS, Toft D. Defining the requirements for Hsp40 and Hsp70 in the Hsp90 chaperone pathway. J Biol Chem. 2006;281: 26235–26244. 10.1074/jbc.M605417200 16854979

[44] Breuer K, Foroushani AK, Laird MR, Chen C, Sribnaia A, Lo R, et al InnateDB: systems biology of innate immunity and beyond—recent updates and continuing curation. Nucleic Acids Res. 2013;41: D1228–33. 10.1093/nar/gks1147 23180781 PMC3531080

[45] Ako-Adjei D, Fu W, Wallin C, Katz KS, Song G, Darji D, et al HIV-1, human interaction database: current status and new features. Nucleic Acids Res. 2015;43: D566–70. 10.1093/nar/gku1126 25378338 PMC4383939

[46] Banerjee S, Liao L, Russo R, Nakamura T, McKercher SR, Okamoto S-I, et al Isobaric tagging-based quantification by mass spectrometry of differentially regulated proteins in synaptosomes of HIV/gp120 transgenic mice: implications for HIV-associated neurodegeneration. Exp Neurol. 2012;236: 298–306. 10.1016/j.expneurol.2012.04.013 22575597 PMC3392539

[47] Plant K, Fairfax BP, Makino S, Vandiedonck C, Radhakrishnan J, Knight JC. Fine mapping genetic determinants of the highly variably expressed MHC gene *ZFP57* . Eur J Hum Genet. 2014;22: 568–571. 10.1038/ejhg.2013.244 24193346 PMC3953924

[48] Fellay J, Ge D, Shianna KV, Colombo S, Ledergerber B, Cirulli ET, et al Common genetic variation and the control of HIV-1 in humans. PLoS Genet. 2009;5: e1000791 10.1371/journal.pgen.1000791 20041166 PMC2791220

[49] Nguyen DG, Yin H, Zhou Y, Wolff KC, Kuhen KL, Caldwell JS. Identification of novel therapeutic targets for HIV infection through functional genomic cDNA screening. Virology. 2007;362: 16–25. 10.1016/j.virol.2006.11.036 17257639

[50] Horton R, Wilming L, Rand V, Lovering RC, Bruford EA, Khodiyar VK, et al Gene map of the extended human MHC. Nat Rev Genet. 2004;5: 889–899. 10.1038/nrg1489 15573121

[51] Manolio TA, Collins FS, Cox NJ, Goldstein DB, Hindorff LA, Hunter DJ, et al Finding the missing heritability of complex diseases. Nature. 2009;461: 747–753. 10.1038/nature08494 19812666 PMC2831613

[52] Rappaport J, Cho YY, Hendel H, Schwartz EJ, Schächter F, Zagury J-F. 32 bp *CCR-5* gene deletion and resistance to fast progression in HIV-1 infected heterozygotes. Lancet. 1997;349: 922–923. 10.1016/S0140-6736(05)62697-9 9093257

[53] Van Manen D, Kootstra NA, Boeser-Nunnink B, Handulle MA, van’t Wout AB, Schuitemaker H. Association of *HLA-C* and *HCP5* gene regions with the clinical course of HIV-1 infection. AIDS. 2009;23: 19–28. 19050382 10.1097/QAD.0b013e32831db247

[54] The 1000 Genomes Project Consortium. An integrated map of genetic variation from 1,092 human genomes. Nature. 2012;491: 56–65. 10.1038/nature11632 23128226 PMC3498066

