# Supplementary material for: Identification of Genes Whose Expression Profile Is Associated with Non-Progression towards AIDS Using eQTLs
Source: PLoS One. 2015 Sep 14;10(9):e0136989. doi: 10.1371/journal.pone.0136989 (PMC4569262; doi:10.1371/journal.pone.0136989)
Supplement: S1 File — Which eQTL database can the functional SNPs traced back to? Q-Q plots for the analyses. Negative results for rapid progression. Interactions between the identified genes and AIDS/HIV. (PDF) [file pone.0136989.s001.pdf]

Supplementary Results

Venn diagram of SNPs

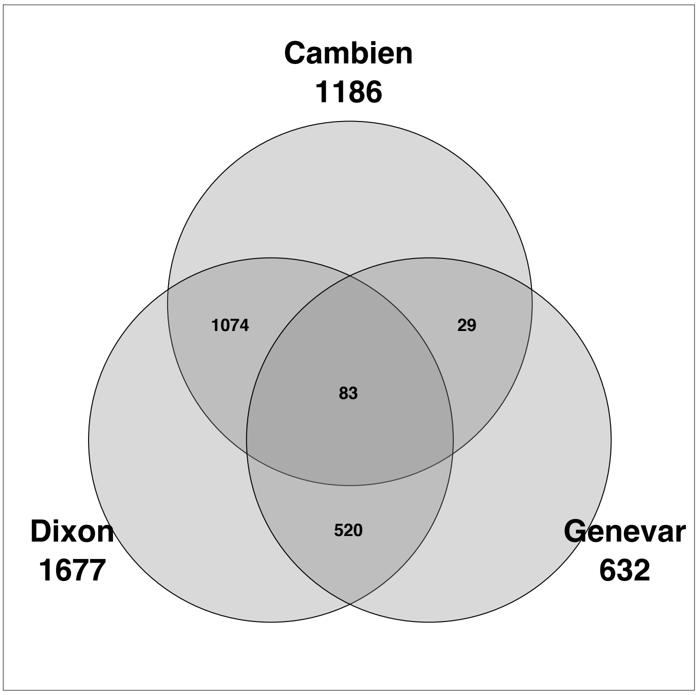

Q-Q plots

GRIV

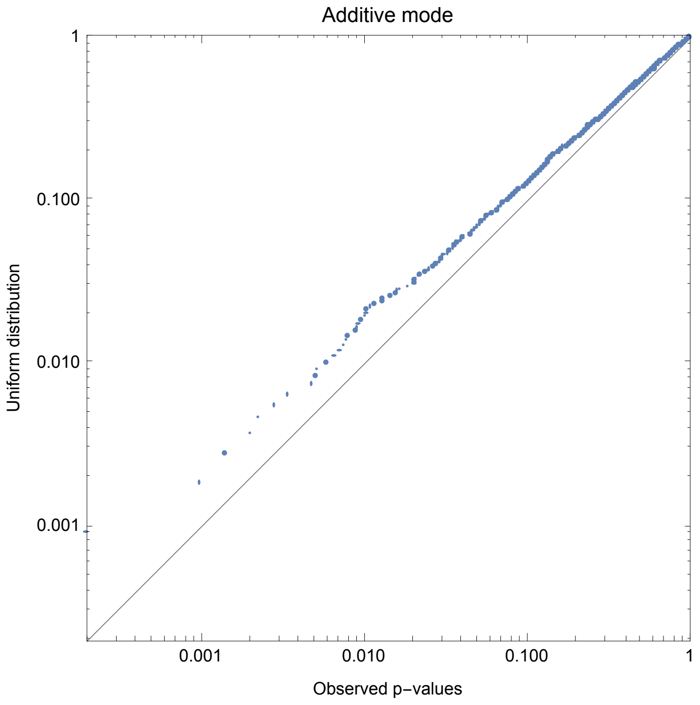

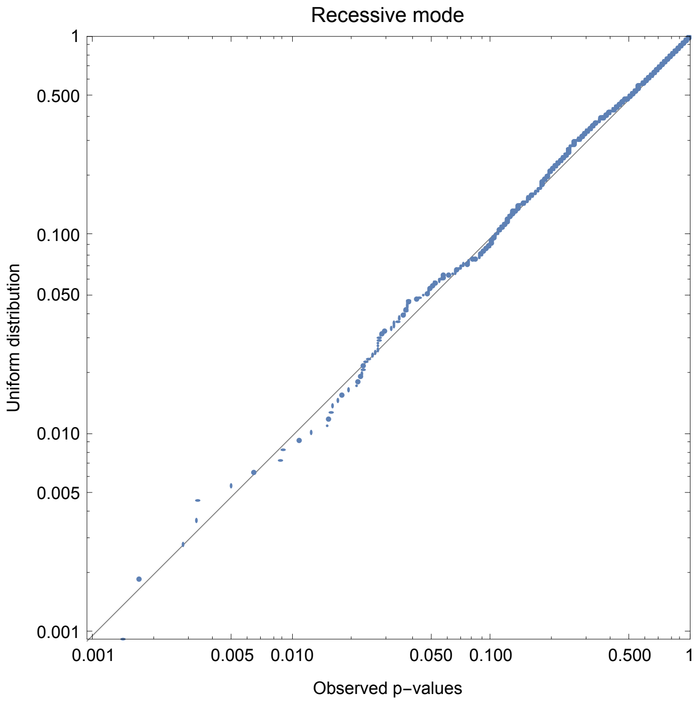

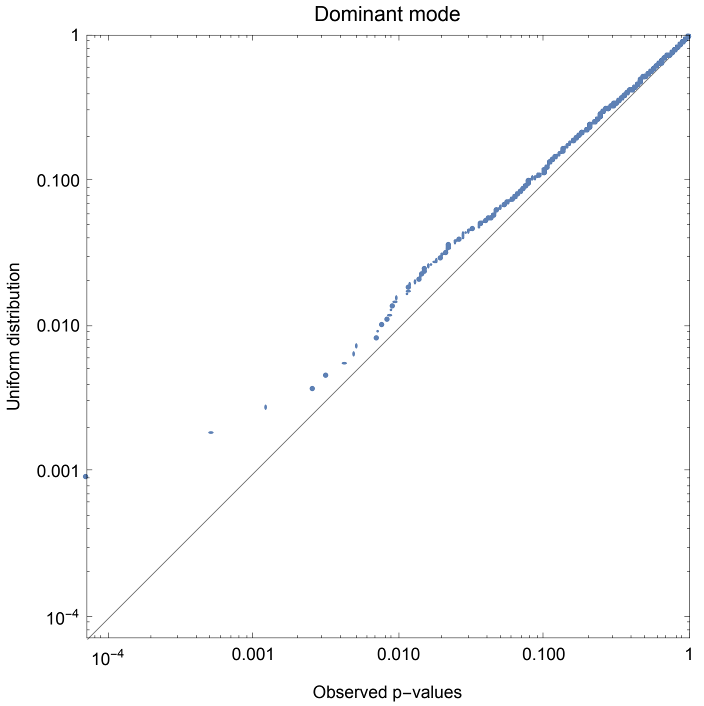
Dominant mode
Uniform distribution
Observed p-values
1
0.100
0.010
0.001
10^{-4}
0.001
0.010
0.100
1

ACS

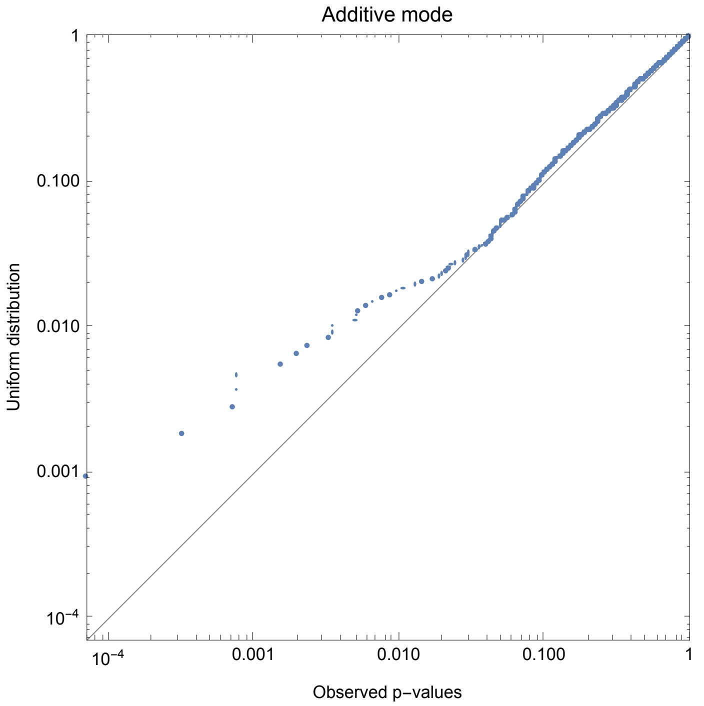
Additive mode
Uniform distribution
Observed p-values
1
0.100
0.010
0.001
10^{-4}
0.001
0.010
0.100
1

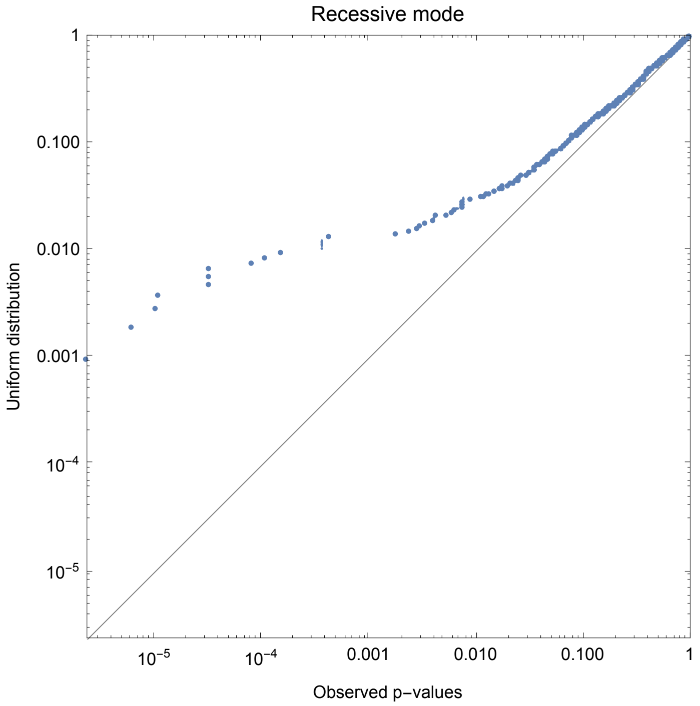

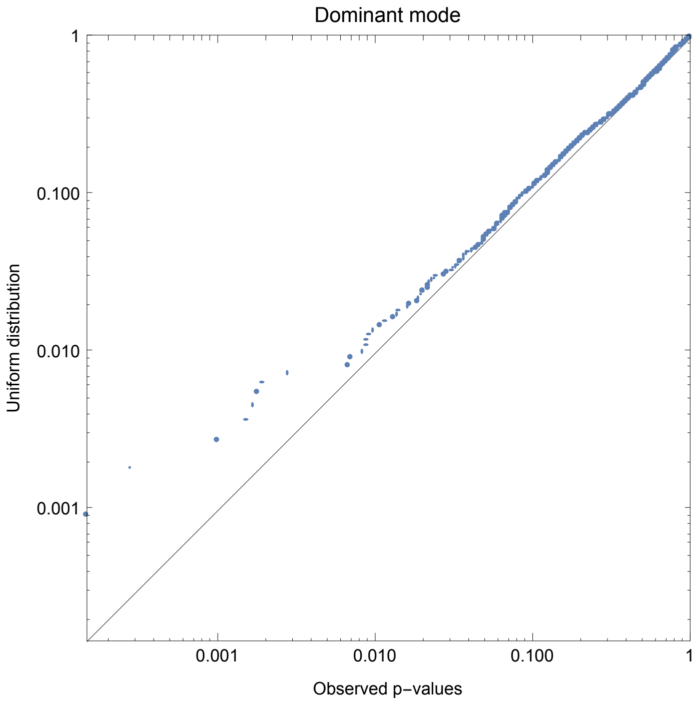

Rapid progression results

The same analysis was carried out to try to identify factors associated with rapid progression to AIDS. The randomisation procedures clearly showed that we did not have significant results: The arrow represents the number of associations we obtained (1) on the histogram of the number of associations obtained from randomisations.

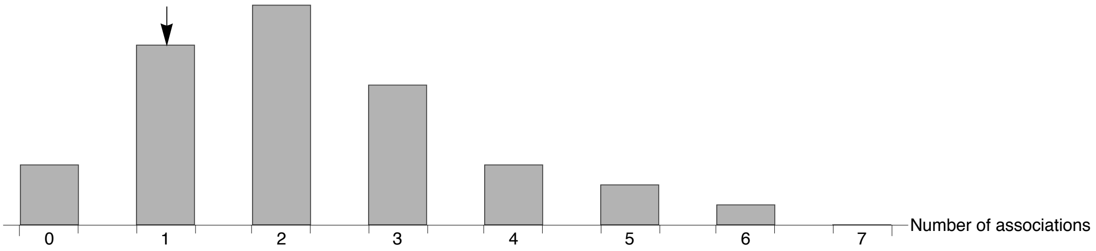

Interactions between candidate genes

Further information gathered using

- http://www.innatedb.com/
- http://string-db.org/
- NCBI's Gene server

Interactions between MRPS10 and HIV-related gene products

MRPS10 interacts with

- C1QBP: Involved in HIV-1 replication, presumably by contributing to splicing of viral RNA.
- BAG3: HIV-1 interactions
  - Replication-independent expression of anti-apoptosis marker genes in human peripheral blood mononuclear cells infected with the wild-type HIV-1 and reverse transcriptase variants.
    Sharma PL, Chunduri H, Wise J, Mindley R, Rimland D.
    Viral Immunol. 2012 Feb;25(1):12-20. doi: 10.1089/vim.2011.0057. Epub 2012 Jan 12.
    PMID: 22239233
  - BAG3 protein regulates caspase-3 activation in HIV-1-infected human primary microglial cells.Rosati A, Khalili K, Deshmane SL, Radhakrishnan S, Pascale M, Turco MC, Marzullo L.
    J Cell Physiol. 2009 Feb;218(2):264-7. doi: 10.1002/jcp.21604.
    PMID: 18821563
  - Evidence for BAG3 modulation of HIV-1 gene transcription.
    Rosati A, Leone A, Del Valle L, Amini S, Khalili K, Turco MC.
    J Cell Physiol. 2007 Mar;210(3):676-83.
    PMID: 17187345
- ILF3
- SDHB
- UBC

DNAJB12

- DSTN: HIV-1 interaction
- B/ EGFR : HIV-1 interaction
- C/ HSPA8: HIV-1 interaction
- D/MME : HIV-1 interaction
- E/ MYC : HIV-1 interaction
- F/ SGTA : HIV-1 interaction
- G/ UBC : HIV-1 interaction

MRPS7

- ACTA2 : HIV-1 interaction
- C1QBP : HIV-1 interaction
- CEP57 : HIV-1 interaction
- EEF1A1 : HIV-1 interaction
- HNRNPAB : HIV-1 interaction
- MRPL54 : HIV-1 interaction
- MRPS9 : HIV-1 interaction
- PSMA5 : HIV-1 interaction
- RNF2 : HIV-1 interaction
- TUBG1

CCT8

- ACTB: HIV-1 interaction
- APP: HIV-1 interaction
- ATP6V1B2: HIV-1 interaction
- CBL: HIV-1 interaction
- CCT2: HIV-1 interaction
- CCT4: HIV-1 interaction
- CCT6A: HIV-1 interaction
- CDC20: HIV-1 interaction
- CDK2: HIV-1 interaction
- CDK5: HIV-1 interaction
- CDK9: HIV-1 interaction
- COPS5: HIV-1 interaction
- CUL1: HIV-1 interaction
- FBXO6: HIV-1 interaction
