# Supplementary material for: Identification of Genes Whose Expression Profile Is Associated with Non-Progression towards AIDS Using eQTLs
Source: PLoS One. 2015 Sep 14;10(9):e0136989. doi: 10.1371/journal.pone.0136989 (PMC4569262; doi:10.1371/journal.pone.0136989)
Supplement: S2 File — Mode in the Cambien dataset. Additional details about the cohorts. Workflow for preprocessing genomic data and testing the association with slow or non-progression. Parameters for linkage disequilibrium. Details about the SNP randomisation. (PDF) [file pone.0136989.s002.pdf]

Supplementary Methods

Sets of functional SNPs

The GHS_Express study corresponds to a cohort of 1490 patients extracted from the Gutenberg Heart Study cohort on cardio-vascular diseases. Monocyte cells were genotyped with the Affymetrix 1M chips and gene expression was measured on 12,808 genes (1). In the Gene Expression Analysis Based on Imputed Genotype database, gene expression was tested in B lymphocyte cells from 206 British families. The expression of 6600 genes was tested on 408,273 SNPs, after genotyping with Illumina 300k chips (2). The Genevar study included data from lymphoblastoid cells genotyped with Illumina 500k chips from 75 unrelated European individuals. Associations have been tested for 394,651 SNPs and 17,945 genes (3-5). In this study we focused on biallelic SNPs and excluded A/T and G/C polymorphisms, due to inconsistencies in the databases.

Mode in the Cambien dataset

The Cambien dataset does not directly provide regulation information (1). Instead, it gives a global ANOVA -value together with average mRNA quantification for all available genotypes AA, AB, BB. In order to ascribe a mode and regulation direction to a SNP, four models (additive, recessive, dominant and overdominant) were fitted and the one that minimised the was considered the appropriate mode of the association.

Each SNP/gene pair data are fitted against one of the following modes:

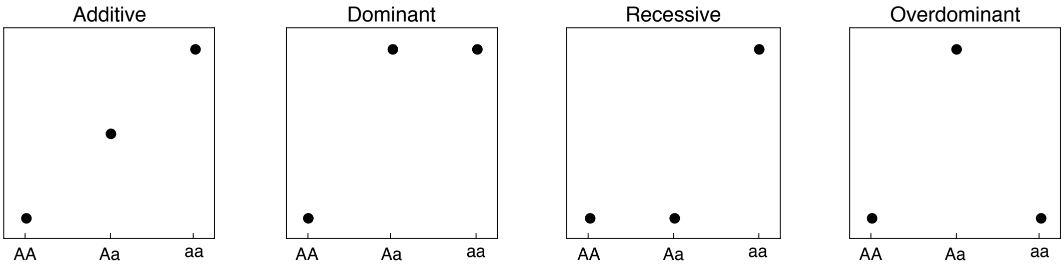

Whichever has the best R^2 value is selected as the mode of association. The overdominant mode is important as a safeguard against poor fits. The R^2 values are calculated thus:

$$\rho_{add} = \frac{(\text{maa} - 2\,\text{mAa} + \text{mAA})^2}{\text{nAA}\,(4\,\text{naa} + \text{nAa}) + \text{naa}\,\text{nAa}}$$

$$\rho_{rec} = \frac{(\text{mAA} - \text{mAa})^2}{\text{naa}\,(\text{nAa} + \text{nAA})}$$

$$\rho_{dom} = \frac{(\text{mAa} - \text{maa})^2}{\text{nAA}\,(\text{naa} + \text{nAa})}$$

$$\rho_{overdom} = \frac{(\text{mAA} - \text{maa})^2}{\text{nAa}\,(\text{naa} + \text{nAA})}$$

Then the direction of the association (lower or higher transcription rate) is given by

$$\sigma_{add} = \text{sgn}(\text{maa naa nAa} + 2\,\text{maa naa nAA} - \text{mAa naa nAa} + \text{mAa nAa nAA} - 2\,\text{mAA naa nAA} - \text{mAA nAa nAA})$$

$$\sigma_{rec} = \text{sgn}(\text{maa nAa} + \text{maa nAA} - \text{mAa nAa} - \text{mAA nAA})$$

$$\sigma_{dom} = \text{sgn}(\text{maa naa} + \text{mAa nAa} - \text{mAA naa} - \text{mAA nAa})$$
$$\rho_{overdom} = \text{NA}$$

When a homozygote is rare (<30), the mode is kept uncertain (eg, additive OR dominant).

Regulation in the Dixon dataset

The Dixon dataset provides regulation information (*p*-value and effect), however, all associations are in the additive mode (2).

Regulation in the Genevar dataset

The Genevar dataset was obtained by performing a linear regression between genotypes and mRNA levels in HapMap 2 (6). All modes were tested and only SNP-gene pairs of SNPs less than 1 Mbp apart were considered.

Cohorts

The Genomics of Resistance to Immunodeficiency Virus Cohort (GRIV)

The subjects were included on the basis of the main clinical outcomes, CD4 T-cell count and time to disease progression. NPs were defined as asymptomatic HIV-1 infected individuals for more than 8 years with no treatment and a CD4 T-cell count above 500 cells/mm^3. DNA was obtained from fresh peripheral blood mononuclear cells or from EBV-transformed cell lines (7).

French control group

DESIR was a 9-year follow-up study designed to clarify the development of the insulin resistance syndrome. Subjects were recruited from 1994 to 1996 from volunteers insured by the French social security system, which offers periodic free-of-charge health examinations (8). This control group comprised 694 participants both non-obese and normoglycemic of the DESIR trial, all French and HIV-1 seronegative. It was composed of 281 males and 413 females aged from 30 to 64.

Processing genomic data

Preprocessing

SNP data were preprocessed using PLINK v1.07 (9). We excluded individuals with relatedness higher than and outliers based on population stratification (farther than six standard deviations from the mean using two principal components). SNPs were excluded when, within the control group, they failed to pass the Hardy-Weinberg test ($p > 5 \times 10^{-6}$), when the minor allelic frequency was less than 1% or when missing data were greater than 2%. Individuals with more than 5% missing data or with high heterozygosity were excluded.

Imputation

In order to identify all the known SNPs in LD with our selected list of SNPs, present in the HapMap database, we imputed all SNPs in the GRIV, ACS and control subjects. For that, we used the ShapeIT software to pre-phase data (10,11), the Impute software version 2.1 (12), and the 1000 Genomes phase I data (13). Only the SNPs reliably imputed (information score P > 0.8) were

retained and the genotype with posterior probability greater than 0.95 was assumed.

Association tests

The SNPs associated with non-progression towards AIDS were sought in the GRIV cohort and then replicated in ACS. In order to be replicated an association must be consistent in terms of its mode and effect direction. (R scripts are available upon request.)

Association with non-progression GRIV

For each functional SNP, we computed the p-values of the association in the GRIV cohort with non-progression or with rapid progression in the GRIV cohort. Two standard case-control analyses were performed: non-progression (NP) vs control and rapid progression (RP) vs control. The statistical analysis was carried through using a logistic regression implemented with GNU R v3.0.2 (14). All modes (dominant, recessive, and additive) were tested. Sex as well as the first two stratification axes as calculated by EIGENSOFT (15) were included as covariates: $\text{logit(NP)} \sim \text{sex} + PC_1 + PC_2 + s$ and $\text{logit(RP)} \sim \text{sex} + PC_1 + PC_2 + s$, where s is the SNP encoded as {0, 1, 2}, {0, 1, 1} and {0, 0, 1} depending on the mode of interaction (add, dom, rec resp.). The significance threshold for an association with progression was set at $\alpha = 0.05$.

Replication in ACS

For each functional SNP, we computed the p-values of the association in the ACS cohort with progression using the (censored) variable 'time to AIDS 1993 after HIV-1 infection' (16) and the Cox regression model (GNU R v3.0.2, library 'survival' v2.37-7). Previous studies had shown that this phenotype was the best one to match the progression phenotypes in the case-control GRIV study (16-21). The first two stratification axes as calculated by EIGENSOFT (15) were included as covariates (sex was not included as all subjects are male): $\log(h) \sim PC_1 + PC_2 + s$ where h is the hazard function.

Linkage disequilibrium

We used the SNAP Pairwise LD calculator with the following parameters

- SNP data set: 1000 Genomes Pilot 1
- Population panel CEU
- Threshold $r^2 \geq 0.2$
- Distance less than 500 kbp (http://www.broadinstitute.org/mpg/snap/ldsearchpw.php)

SNP randomisation

In order to avoid any bias, sets of SNPs comparable to the set of functional SNPs described above were selected for the SNP randomisation. Other factors (sex, stratification principal components, outcome) were maintained unaltered. The sets of SNPs had to be comparable in terms of (a) the number of SNPs that are genotyped/imputed, (b) the minor allele frequency in the European populations (bins 0.03, 0.05, 0.1, 0.2, 0.5), (c) the distribution of the number of SNPs in complete linkage disequilibrium in Hapmap (bins 1, 2, 5, 10, 10+) and (d) the distribution of distances to the nearest genes (bins intragenic, 1, 1000, 20,000, 20,000+). (e) SNPs were drawn from a set of SNPs for which genomic data were of high quality (posterior probability of current imputation greater than 0.98). (f) Linkage disequilibrium was avoided using SNAP Pairwise LD as detailed above. The

constraints (a-d) being rather conservative, we ended up with 24 sets of random SNPs. The (f) step added some uncertainty to the final size of the random SNP sets, which contained between 636 and 652 SNPs.

References

1. Zeller T, Wild P, Szymczak S, Rotival M, Schillert A, Castagne R, et al. Genetics and beyond--the transcriptome of human monocytes and disease susceptibility. 2010; 5(5):e10693.
2. Dixon AL, Liang L, Moffatt MF, Chen W, Heath S, Wong KCC, et al. A genome-wide association study of global gene expression. Nat Genet. Nature Publishing Group; 2007; 39(10):1202-1207.
3. Dimas AS, Deutsch S, Stranger BE, Montgomery SB, Borel C, Attar-Cohen H, et al. Common regulatory variation impacts gene expression in a cell type-dependent manner. Science. 2009; 325(5945):1246-1250.
4. Stranger BE, Forrest MS, Clark AG, Minichiello MJ, Deutsch S, Lyle R, et al. Genome-Wide Associations of Gene Expression Variation in Humans. PLoS Genet. 2005; 1(6):e78.
5. Stranger BE, Forrest MS, Dunning M, Ingle CE, Beazley C, Thorne N, et al. Relative impact of nucleotide and copy number variation on gene expression phenotypes. Science. American Association for the Advancement of Science; 2007; 315(5813):848-853.
6. International HapMap Consortium. The International HapMap Project. Nature. 2003; 426(6968):789-796.
7. Maher B. Personal genomes: The case of the missing heritability. Nature. 2008; :18-21.
8. Balkau B. [An epidemiologic survey from a network of French Health Examination Centres, (D.E.S.I.R.): epidemiologic data on the insulin resistance syndrome]. Rev Epidemiol Sante Publique. 1996; 44(4):373-375.
9. Purcell S, Neale B, Todd-Brown K, Thomas L, Ferreira MAR, Bender D, et al. PLINK: a tool set for whole-genome association and population-based linkage analyses. Am J Hum Genet. 2007; 81(3):559-575.
10. Delaneau O, Marchini J, Zagury J-F. A linear complexity phasing method for thousands of genomes. Nat Methods. 2012; 9(2):179-181.
11. Delaneau O, Zagury J-F, Marchini J. Improved whole-chromosome phasing for disease and population genetic studies. Nat Methods. 2013; 10(1):5-6.
12. Marchini J, Howie B, Myers S, McVean G, Donnelly P. A new multipoint method for genome-wide association studies by imputation of genotypes. Nat Genet. 2007; 39(7):906-913.
13. The 1000 Genomes Project Consortium. An integrated map of genetic variation from 1,092 human genomes. Nature. Nature Publishing Group; 2012; 491(7422):56-65.
14. R Core Team. R: A language and environment for statistical computing [Internet]. 3rd ed. 2014. Available from: http://www.R-project.org/
15. Price AL, Patterson NJ, Plenge RM, Weinblatt ME, Shadick NA, Reich D. Principal components analysis corrects for stratification in genome-wide association studies. Nat Genet [Internet]. 2006; 38(8):904-909. Available from: http://eutils.ncbi.nlm.nih.gov/entrez/eutils/elink.fcgi?dbfrom=pubmed&id=16862161&retmode=ref&cmd=prlinks

16. Winkler CA, Hendel H, Carrington M, Smith MW, Nelson GW, O Brien SJ, et al. Dominant Effects of CCR2-CCR5 Haplotypes in HIV-1 Disease Progression. JAIDS Journal of Acquired Immune Deficiency Syndromes. 2004; 37(4):1534-1538.

17. Carrington M. HLA and HIV-1: Heterozygote Advantage and B*35-Cw*04 Disadvantage. Science. 1999; 283(5408):1748-1752.

18. Hendel H, Caillat-Zucman S, Lebuanec H, Carrington M, O'Brien S, Andrieu JM, et al. New class I and II HLA alleles strongly associated with opposite patterns of progression to AIDS. J Immunol. 1999; 162(11):6942-6946.

19. Limou S, Coulonges C, Herbeck JT, Van Manen D, An P, Le Clerc S, et al. Multiple-Cohort Genetic Association Study Reveals CXCR6 as a New Chemokine Receptor Involved in Long-Term Nonprogression to AIDS. J Infect Dis. 2010; 202(6):908-915.

20. Dean M, Carrington M, Winkler C, Huttley GA, Smith MW, Allikmets R, et al. Genetic Restriction of HIV-1 Infection and Progression to AIDS by a Deletion Allele of the CKR5 Structural Gene. Science. 1996; 273(5283):1856-1862.

21. Hendel H, Hénon N, Lebuanec H, Lachgar A, Poncelet H, Caillat-Zucman S, et al. Distinctive effects of CCR5, CCR2, and SDF1 genetic polymorphisms in AIDS progression. J Acquir Immune Defic Syndr Hum Retrovirol. 1998; 19(4):381-386.
